# Association Between the Implementation of Hospital-Based Palliative Care and Use of Intensive Care During Terminal Hospitalizations

**DOI:** 10.1001/jamanetworkopen.2019.18675

**Published:** 2020-01-08

**Authors:** May Hua, Yewei Lu, Xiaoyue Ma, R. Sean Morrison, Guohua Li, Hannah Wunsch

**Affiliations:** 1 Department of Anesthesiology, Columbia University College of Physicians and Surgeons, New York, New York; 2 Department of Epidemiology, Columbia University Mailman School of Public Health, New York, New York; 3 Center for Health Policy and Outcomes in Anesthesia and Critical Care, Department of Anesthesiology, Columbia University College of Physicians and Surgeons, New York, New York; 4 Brookdale Department of Geriatrics and Palliative Medicine, Icahn School of Medicine at Mount Sinai, New York, New York; 5 Department of Critical Care Medicine, Sunnybrook Health Sciences Center, Toronto, Ontario, Canada; 6 Department of Anesthesia and Interdisciplinary Department of Critical Care Medicine, University of Toronto, Toronto, Ontario, Canada

## Abstract

**Importance:**

The use of intensive care at the end of life continues to be common. Although the provision of palliative care has been advocated as a way to mitigate the use of high-intensity care, it is unknown whether implementation of hospital-based palliative care services is associated with reduced use of intensive care at the end of life.

**Objective:**

To determine whether implementation of hospital-based palliative care services is associated with decreased intensive care unit (ICU) use during terminal hospitalizations.

**Design, Setting, and Participants:**

This cohort study included 51 hospitals in New York State that either did or did not implement a palliative care program between 2008 and 2014. Hospitals that consistently had a palliative care program during the study period were excluded. Participants were adult patients who died during hospitalization. Data analysis was performed between January 2018 and July 2019.

**Exposure:**

Implementation of a palliative care program.

**Main Outcomes and Measures:**

The primary outcome was ICU use. A difference-in-differences analysis was performed using multilevel regression to assess the association between implementing a palliative care program and ICU use during terminal hospitalizations while adjusting for patient and hospital characteristics and time trends.

**Results:**

During the study period, 73 370 patients (mean [SD] age, 76.5 [14.1] years; 38 467 [52.4%] women) died during hospitalization, of whom 37 628 (51.3%) received care in hospitals that implemented palliative care services and 35 742 (48.7%) received care in a hospital without palliative care implementation. Patients who received care in hospitals after implementation of palliative care services were less likely to receive intensive care than patients admitted to the same hospitals before implementation (49.3% vs 52.8%; difference 3.5%; 95% CI, 2.5%-4.5%; *P* < .001). Compared with hospitals that never had a palliative care program, the implementation of palliative care was associated with a 10% reduction in ICU use during terminal hospitalizations (adjusted relative risk, 0.90; 95% CI, 0.85-0.95; *P* < .001).

**Conclusions and Relevance:**

The implementation of hospital-based palliative care services in New York State was associated with a modest reduction in ICU use during terminal hospitalizations.

## Key Points

**Question:**

Is implementation of palliative care services associated with a decrease in the use of intensive care at the end of life for hospitalized patients?

**Findings:**

In this cohort study, implementation of palliative care at a hospital was associated with a 10% reduction in intensive care unit use for 73 370 patients who died during their hospitalization.

**Meaning:**

Implementation of hospital-based palliative care services may be associated with decreased treatment intensity at the end of life for hospitalized patients.

## Teaser

This cohort study examines whether implementation of hospital-based palliative care services is associated with decreased intensive care unit use during terminal hospitalizations.

## Introduction

High-intensity care at the end of life has the potential to be discordant with patient preferences and nonbeneficial.^1,2,3^ Therefore, both the use of intensive care during the last 30 days of life and dying in an acute care setting are considered negative indicators of the quality of end-of-life care.^4^ Furthermore, although the percentage of patients dying at home and the percentage of patients who use hospice have increased over time, the use of intensive care services within the last month of life has also increased, suggesting that the use of different types of supportive care measures at the end of life may simply be increasing.^5^ Several organizations, including the Institute of Medicine and the Choosing Wisely Campaign,^6,7,8^ have advocated increasing the use of palliative care to mitigate high treatment intensity and ameliorate the discrepancy between the care that patients desire and the care they actually receive. Although palliative care programs have continued to expand nationally,^9^ it is not known whether this growth is associated with decreases in treatment intensity at the end of life. Thus, the goal of this study was to assess whether the implementation of hospital-based palliative care services is associated with a subsequent decrease in the use of intensive care during terminal hospitalizations.

## Methods

### Patients and Data Collection

The study protocol was reviewed and approved by the institutional review board of Columbia University Medical Center. Written informed consent was waived. Because the data were deidentified, the research was judged to involve minimal risk to participants, and the waiver would not adversely affect the rights and welfare of the participants. This cohort study adheres to the Strengthening the Reporting of Observational Studies in Epidemiology (STROBE) reporting guideline.^10^

Data for this study came from a patient-level database from the New York Statewide Planning and Research Cooperative System and a collection of hospital-level characteristics from the American Hospital Association Annual Survey; information about palliative care programs came from the National Palliative Care Registry for the years 2008 to 2014. Details on the creation of this data set have been published previously.^11^ Data analyses for this study were performed between January 2018 and July 2019.

The primary exposure for this study was implementation of a hospital-based palliative care program. We chose to use this approach for several reasons: (1) the use of a patient-level exposure may be more subject to indication bias than a hospital-level exposure; (2) with a patient-level exposure, we would not have information about the timing of palliative care consultation, which may make the results difficult to interpret in terms of temporality; and (3) patient-level receipt of palliative care cannot accurately be determined from population-level data at this time.^12^ We included hospitals that developed a palliative care program during the study period, as well as hospitals that never had a palliative care program as controls. Hospitals that consistently had a palliative care program throughout the study period were excluded. To reduce misclassification, for hospitals that implemented a palliative care program during the study period, we excluded data from that hospital for the year during which the program was established and for the subsequent year, both to allow for penetration of the program within the hospital and because we did not have information about when during the year the program was initiated. In addition, hospitals that developed a palliative care program during the first year of the study period (2008) or the last 2 years (2013 and 2014) were excluded, because these hospitals would not have enough baseline or follow-up data, respectively. We also excluded small hospitals (<100 beds) and rural hospitals to reduce heterogeneity in hospital characteristics, as well as hospitals where less than 1% of all admissions annually received intensive care, averaged over the study period.

We included all patients aged 18 years or older who died during a terminal hospitalization in New York State in the relevant hospitals. We excluded patients with multiple death records or with missing identifiers. Patient-level covariates that were available within the Statewide Planning and Research Cooperative System included age, sex, race, insurance type, urban residence, patient type (nonsurgical or surgical), number of Elixhauser comorbidities,^13^ diagnosis of sepsis,^14^ risk of mortality, and year of admission. The risk of mortality indicator within the Statewide Planning and Research Cooperative System is calculated using a proprietary grouping software developed by 3M Health Information Systems that is built into the data set and is based on age, comorbidities, procedures, and principal diagnosis for the hospitalization. Hospital-level variables from the American Hospital Association Annual Survey included teaching status of the hospital, hospital bed number, total number of admissions, number of surgical procedures, and number of physician and nurse full-time equivalents, adjusted for hospital bed number; variables were matched using American Hospital Association data corresponding to the patient’s year of admission (for further details on covariates, see the eAppendix in the Supplement).

### Outcomes

The primary outcome for this study was receipt of intensive care, defined as receiving care in an intensive care unit (ICU) (based on having a charge for ICU bed utilization). Patients with intermediate ICU charges were not classified as having received intensive care. Secondary outcomes included hospital length of stay, use of dialysis, days in the ICU (based on number of ICU bed utilization charges), and use of mechanical ventilation for patients admitted to the ICU during their hospitalization.

### Statistical Analysis

We summarized characteristics of hospitals that did and did not implement palliative care; we also summarized demographic and clinical characteristics and calculated standardized differences for patients who received care in hospitals with and without palliative care programs. We assessed the association between implementation of hospital-based palliative care and outcomes using a difference-in-differences approach and multilevel regression, modeling hospital as a random effect. The difference-in-differences model compares the change in outcomes before and after implementation of palliative care in hospitals that instituted a program with the change in outcomes in hospitals that never had a palliative care program over the same period; this model estimates the association between implementing a palliative care program and patient outcomes during terminal hospitalizations, adjusting for secular trends. To account for the fact that hospitals did not all implement palliative care at the same time, we specified a model that allows for variation in the timing of an intervention (see eAppendix in the Supplement for further details).^15^ The parallel trend assumption was checked for all outcomes using visual inspection (eFigure in the Supplement). For all analyses, logistic regression was used to model uncommon binary outcomes (ie, those occurring with frequency <10%), Poisson regression with robust error variance was used for common binary outcomes (ie, those occurring with a frequency ≥10%),^16^ and negative binomial regression was used for ordinal outcomes. Because the primary exposure of interest was a hospital-level variable, we performed grand-mean centering for patient-level covariates.^17^ In regression models, we included individual patient age, sex, race, and risk of mortality during hospitalization a priori as confounders, as well as year of admission and any additional patient or hospital variables that had a standardized difference greater than 0.1.^18^ For the primary analysis, 2-sided *P* < .05 was considered as statistically significant. The χ^2^ test was used to test the unadjusted difference between ICU use before and after implementation in the implementation hospitals. Given the number of outcomes tested in secondary analyses, we used a Bonferroni correction to reduce the likelihood of type I error, resulting in the threshold for statistical significance being set at *P* = .002.

### Secondary Analyses

A priori, we planned several secondary analyses. To address large differences in hospital characteristics, we repeated the difference-in-differences analysis, stratifying by teaching status and hospital bed number. Also, because we used a hospital-level exposure to approximate patient-level receipt of specialized palliative care, we repeated our analysis in a subgroup analysis of patients with a diagnosis of metastatic cancer (identified using the coding algorithm from the Elixhauser comorbidity index),^13^ because this represents a patient population that is potentially more likely to have received a palliative care consultation during the hospital stay, if these services were available. Finally, we performed a post hoc analysis, adjusting for the number of ICU beds within the hospital, given evidence that use of intensive care may vary depending on supply.^3,19^ The number of ICU beds was ascertained using data from the American Hospital Association Annual Survey for each hospital for each year; patients cared for in hospitals for which this information was not available were excluded from this analysis (for details, see eAppendix in the Supplement). Database management and statistical analysis were performed using SAS statistical software version 9.4 (SAS Institute) and Stata statistical software version 13.1 (StataCorp).

## Results

### Hospital and Patient Characteristics

Within New York State from 2008 to 2014, 24 hospitals implemented a program during the study period and 27 never had a program; there were 83 hospitals that consistently had a palliative care program for all years and were excluded. Hospitals that implemented hospital-based palliative care were more likely to have teaching status compared with hospitals that never had a program (14 hospitals [58.3%] vs 11 hospitals [40.7%]), to have 400 or more beds (5 hospitals [20.8%] vs 2 hospitals [7.4%]), and to have higher numbers of yearly admissions (median [interquartile range {IQR}], 13 161 [8427-19 102] admissions vs 7761 [4920-11 171] admissions), surgical operations (median [IQR], 8465 [6048-13 594] operations vs 7018 [4181-8654] operations), and fully employed physicians (median [IQR], 27 [8-59] physicians vs 22 [7-49] physicians) and nurses (median [IQR], 397 [251-591] nurses vs 244 [161-411] nurses) (Table 1). In these hospitals, 73 370 patients (mean [SD] age, 76.5 [14.1] years; 38 467 women [52.4%]) had a hospitalization during which they died (Figure); 37 628 patients (51.3%) received care in hospitals that implemented palliative care, and 35 742 (48.7%) received care in a hospital without implementation. For patients in the implementation hospitals, 17 146 patients (45.6%) received care before implementation, and 20 482 (54.5%) received care after implementation. Overall, patients cared for in hospitals that implemented palliative care were younger (mean [SD] age, 75.7 [14.5] years vs 77.4 [13.6] years) and more likely to be of black race (6355 patients [16.9%] vs 3229 patients [9.0%]), live in an urban area (22 362 patients [59.4%] vs 16 202 patients [45.3%]), have private insurance (8426 patients [22.4%] vs 6250 patients [17.5%]), and receive a diagnosis of sepsis during their hospitalization (20 878 patients [55.5%] vs 17 877 patients [50.0%]) (Table 2).

**Table 1.** Characteristics of Hospitals in New York State, Stratified by Implementation of a Hospital-Based Palliative Care Program

Characteristic	Implementation of Hospital-Based Palliative Care, Hospitals, No. (%)	
	No (n = 27)	Yes (n = 24)
Teaching hospital^a^	11 (40.7)	14 (58.3)
Beds, No.^b^		
100-399	25 (92.6)	19 (79.2)
≥400	2 (7.4)	5 (20.8)
Overall hospital admissions with intensive care, median (IQR), %^b^	10 (7-14)	9 (7-12)
1 to <5	1 (3.7)	2 (8.3)
5 to <7	6 (22.2)	3 (12.5)
7 to <10	4 (14.8)	9 (37.5)
10 to <15	11 (40.7)	8 (33.3)
15 to <20	1 (3.7)	0
≥20	4 (14.8)	2 (8.3)
Total admissions, median (IQR), No.^b^	7761 (4920-11 171)	13 161 (8427-19 102)
Total surgical operations, median (IQR), No.^b^	7018 (4181-8654)	8465 (6048-13 594)
Full-time equivalent physicians and dentists, median (IQR), No.^b^	22 (7-49)	27 (8-59)
Full-time equivalent registered nurses, median (IQR), No.^b^	244 (161-411)	397 (251-591)

Abbreviation: IQR, interquartile range.

^a^ Because data were matched by hospital year, several hospitals had characteristics change during the study period. For the purposes of the table, each hospital was assigned on the basis of the category to which they belonged for most years of the study period.

^b^ Because data were matched by hospital year, hospitals had varying values by year. For the purposes of the table, values were averaged over the study period and assigned to each hospital before calculating summary statistics.

**Figure.**
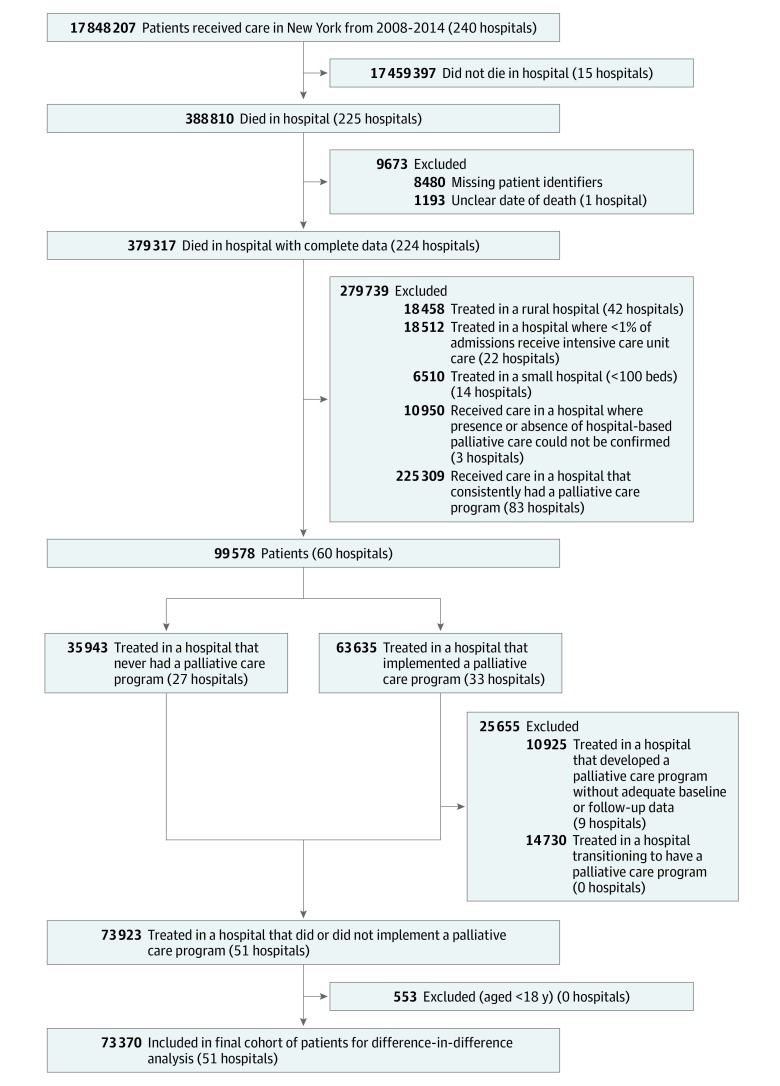
Flowchart of Cohort Creation Flowchart shows inclusion and exclusion criteria for hospitals and patients in the study cohort.

**Table 2.** Baseline Characteristics of Patients Who Died During Hospitalization in Hospitals That Did and Did Not Implement Hospital-Based Palliative Care

Characteristic	Implementation of Hospital-Based Palliative Care, Patients, No. (%)		Standardized Difference
	No (n = 35 742)	Yes (n = 37 628)	
Age, mean (SD), y	77.4 (13.6)	75.7 (14.5)	
Age range, y			
18-64	6169 (17.3)	8063 (21.4)	0.12
65-74	6278 (17.6)	6903 (18.4)	
75-84	10 595 (29.6)	10 616 (28.2)	
≥85	12 700 (35.5)	12 046 (32.0)	
Sex			
Female	18 880 (52.8)	19 587 (52.1)	–0.02
Male	16 862 (47.2)	18 041 (48.0)	
Race			
White	27 386 (76.6)	25 362 (67.0)	0.25
Black	3229 (9.0)	6355 (16.9)	
Other	5019 (14.0)	5626 (15.0)	
Rural residence			
Urban	16 202 (45.3)	22 362 (59.4)	0.33
Mixed	17 176 (48.1)	14 363 (38.2)	
Rural	2224 (6.2)	731 (1.9)	
Insurance			
Medicare	24855 (69.5)	25 216 (67.0)	0.20
Medicaid	1492 (4.2)	1977 (5.3)	
Private	6250 (17.5)	8426 (22.4)	
Self-pay	2532 (7.1)	1306 (3.5)	
Other	613 (1.7)	703 (1.9)	
Surgical	5088 (14.2)	6506 (17.3)	0.08
No. of Elixhauser comorbidities			
0	1018 (2.9)	1246 (3.3)	0.04
1-3	15 076 (42.2)	16 327 (43.4)	
≥4	19 648 (55.0)	20 055 (53.3)	
Risk of mortality at start of hospitalization^a^			
Minor	485 (1.4)	565 (1.5)	0.096
Moderate	3310 (9.3)	3071 (8.2)	
Major	10 922 (30.6)	10 172 (27.0)	
Extreme	21025 (58.8)	23 820 (63.3)	
Sepsis^b^	17 877 (50.0)	20 878 (55.5)	0.11

^a^ The risk of mortality indicator within the New York Statewide Planning and Research Cooperative System is calculated using a proprietary grouping software developed by 3M Health Information Systems, and is based on age, comorbidities, procedures, and principal diagnosis for the hospitalization.

^b^ Patients were identified as having sepsis using the definition described by Angus et al.^14^

### Palliative Care Implementation and ICU Admission

Admission to an ICU during a terminal hospitalization was frequent (50.9% for patients cared for in hospitals that implemented hospital-based palliative care vs 46.0% for patients in hospitals that did not). Patients who received care in a hospital after implementation of palliative care were less likely to receive intensive care than patients in those same hospitals before implementation (49.3% after vs 52.8% before; difference 3.5%; 95% CI, 2.5%-4.5%; *P* < .001). Compared with hospitals that never had a palliative care program, the implementation of palliative care was associated with a 10% reduction in ICU use during terminal hospitalizations (adjusted relative risk [aRR], 0.90; 95% CI, 0.85-0.95; *P* < .001) (Table 3). The implementation of hospital-based palliative care was not associated with significant differences in hospital length of stay (median [IQR], 6 [2-13] days vs 7 [2-15] days; adjusted rate ratio, 1.03; 95% CI, 1.00-1.06) or use of dialysis (8.8% of patients vs 9.4% of patients; adjusted odds ratio, 0.94; 95% CI, 0.82-1.07), or with differences in ICU days (median [IQR], 4 [2-9] days vs 5 [2-10] days; adjusted rate ratio, 0.96; 95% CI, 0.91-1.01) or use of mechanical ventilation (68.5% of patients vs 69.2% of patients; adjusted relative risk, 0.96; 95% CI, 0.92-1.01) for patients admitted to the ICU (Table 3).

**Table 3.** Primary Analysis: Difference-in-Differences Analysis Assessing the Association Between Implementation of Hospital-Based Palliative Care and Resource Utilization During Terminal Hospitalizations

Outcome	Implementation of Hospital-Based Palliative Care, Unadjusted Outcomes			Difference-in-Differences Estimator (95% CI)^a^	*P* Value
	Yes (n = 37 628)		No (n = 35 742)		
	Before	After			
Primary outcome, ICU use, %^b^	52.8	49.3	46.0	0.90 (0.85-0.95)	<.001
Secondary outcomes					
Dialysis, %^c^	9.4	8.8	7.0	0.94 (0.82-1.07)	.34
Hospital length of stay, median (IQR), d^d^	7 (2-15)	6 (2-13)	6 (2-13)	1.03 (1.00-1.06)	.09
Mechanical ventilation, %^b^^,^^e^	69.2	68.5	59.5	0.96 (0.92-1.01)	.12
ICU bed utilization, median (IQR), d^d^^,^^e^	5 (2-10)	4 (2-9)	4 (1-8)	0.96 (0.91-1.01)	.09

Abbreviations: ICU, intensive care unit; IQR, interquartile range.

^a^ This column reports the relative risk, odds ratio, or incidence rate ratio as appropriate. All models are adjusted for age, sex, race, type of insurance, urban residence, risk of mortality during hospitalization, sepsis, year of admission, and hospital characteristics, including teaching hospital, hospital bed number, total admissions per year per total number of beds, total number of surgical operations performed per total number of beds, full-time equivalent physicians per total number of beds, sepsis, and ICU volume.

^b^ Results of multilevel robust Poisson regression, with hospital as a random effect.

^c^ Results of multilevel logistic regression, with hospital as a random effect.

^d^ Results of multilevel negative binomial regression, with hospital as a random effect.

^e^ Only includes patients who died in ICU.

### Secondary Analyses

For analyses stratified by hospital characteristics, the association between implementing hospital-based palliative care and ICU admission was confined to teaching hospitals (aRR, 0.81; 95% CI, 0.74-0.88; *P* < .001) and large hospitals with 400 beds or more (aRR, 0.88; 95% CI, 0.83-0.94; *P* < .001). Implementing palliative care was also associated with a decrease in the number of ICU days for patients admitted to the ICU in teaching hospitals (aRR, 0.87; 95% CI, 0.82-0.93; *P* < .001) and large hospitals with 400 beds or more (aRR, 0.78; 95% CI, 0.66-0.91; *P* = .002) (Table 4).

**Table 4.** Secondary Analyses: Difference-in-Differences Analysis Examining the Association Between Implementing Hospital-Based Palliative Care and Resource Utilization During Terminal Hospitalizations, Stratified by Hospital Characteristics and for Patients With Metastatic Cancer

Outcome	Hospital Type				Bed Number				Patients With Metastatic Cancer (n = 10 177)	
	Nonteaching (n = 28 777)		Teaching (n = 44 593)		100-399 (n = 54 621)		≥400 (n = 18 749)			
	Estimator (95% CI)^a^	*P* Value^b^	Estimator (95% CI)^c^	*P* Value^b^	Estimator (95% CI)^d^	*P* Value^b^	Estimator (95% CI)^e^	*P* Value^b^	Estimator (95% CI)^f^	*P* Value^b^
Primary outcome, ICU use, %^g^	1.00 (0.89-1.13)	.96	0.81 (0.74-0.88)	<.001	0.95 (0.88-1.03)	.18	0.88 (0.83-0.94)	<.001	0.76 (0.65-0.88)	<.001
Secondary outcomes										
Dialysis, %^h^	0.89 (0.66-1.19)	.44	0.89 (0.75-1.05)	.16	1.00 (0.85-1.17)	.97	0.67 (0.45-1.00)	.049	0.86 (0.55-1.34)	.50
Length of stay, median (IQR), d^i^	1.13 (1.07-1.20)	<.001	0.95 (0.90-1.00)	.03	1.03 (0.99-1.08)	.10	0.98 (0.87-1.11)	.81	1.01 (0.93-1.10)	.85
Mechanical ventilation, %^g^^,^^j^	0.88 (0.65-1.18)	.38	0.98 (0.93-1.03)	.47	0.98 (0.93-1.04)	.56	1.00 (0.92-1.09)	.98	0.87 (0.62-1.21)	.41
ICU bed utilization, median (IQR), d^i^^,^^j^	1.11 (1.02-1.20)	.01	0.87 (0.82-0.93)	<.001	0.98 (0.93-1.04)	.58	0.78 (0.66-0.91)	.002	0.93 (0.81-1.06)	.29

Abbreviations: ICU, intensive care unit; IQR, interquartile range.

^a^ This column reports the relative risk, odds ratio, or incidence rate ratio as appropriate. All models are adjusted for age, sex, race, type of insurance, risk of mortality during hospitalization, sepsis, urban residence, year of admission, and hospital characteristics, including hospital bed number, total admissions per year per total number of beds, total number of surgical operations performed per total number of beds, full-time equivalent nurses per total number of beds, and ICU volume.

^b^ After applying a Bonferroni correction, the threshold for statistical significance was considered to be *P* = .002.

^c^ This column reports the relative risk, odds ratio, or incidence rate ratio as appropriate. All models are adjusted for age, sex, race, type of insurance, risk of mortality during hospitalization, sepsis, urban residence, year of admission, and hospital characteristics, including hospital bed number, total admissions per year per total number of beds, total number of surgical operations performed per total number of beds, and ICU volume.

^d^ This column reports the relative risk, odds ratio, or incidence rate ratio as appropriate. All models are adjusted for age, sex, race, type of insurance, urban residence, risk of mortality during hospitalization, sepsis, year of admission, and hospital characteristics, including total admissions per year per total number of beds, total number of surgical operations performed per total number of beds, full-time equivalent physicians per total number of beds, full-time equivalent nurses per total number of beds, and ICU volume.

^e^ This column reports the relative risk, odds ratio, or incidence rate ratio as appropriate. All models are adjusted for age, sex, race, type of insurance, urban residence, risk of mortality during hospitalization, sepsis, year of admission, and hospital characteristics, including teaching hospital, total admissions per year per total number of beds, total number of surgical operations performed per total number of beds, full-time equivalent physicians per total number of beds, full-time equivalent nurses per total number of beds, and ICU volume.

^f^ This column reports the relative risk, odds ratio, or incidence rate ratio as appropriate. All models are adjusted for age, sex, race, type of insurance, urban residence, risk of mortality during hospitalization, sepsis, year of admission, and hospital characteristics, including teaching hospital, hospital bed number, total admissions per year per total number of beds, total number of surgical operations performed per total number of beds, full-time equivalent physicians per total number of beds, full-time equivalent nurses per total number of beds, and ICU volume.

^g^ Results of multilevel robust Poisson regression, with hospital as a random effect.

^h^ Results of multilevel logistic regression, with hospital as a random effect.

^i^ Results of multilevel negative binomial regression, with hospital as a random effect.

^j^ Only includes patients who died in ICU.

For the subgroup of 10 177 critically ill patients with metastatic cancer, there was again an association between implementation of hospital-based palliative care and a significant decrease in ICU use during terminal hospitalizations (adjusted odds ratio, 0.76; 95% CI, 0.65-0.88; *P* < .001) and no significant differences in secondary outcomes (Table 4). We repeated the primary analysis, taking the number of ICU beds at the hospital into account. After excluding patients without information on the number of ICU beds, we included 71 506 patients from 48 hospitals in the analysis. After adjustment for the number of ICU beds at a hospital, the results were unchanged (eTable in the Supplement).

## Discussion

Using a difference-in-differences approach, we found that implementation of a hospital-based palliative care program was associated with a small but measurable decrease in the use of intensive care during terminal hospitalizations. We found similar results for patients with metastatic cancer, a subgroup of patients who may have been likely to receive palliative care consultation, because clinicians may be more likely to recognize that these patients have a terminal illness with poor prognosis, and because there is evidence to support of the benefit of palliative care in patients with cancer.^20^ Although the observed difference was small (absolute decrease of 3.5%), on a population level, the association may be magnified. Approximately 2.8 million people died in the United States in 2017,^21^ of whom 15% are estimated to have died in the ICU.^22^ An ICU stay has an estimated median cost of $10 811 dollars for patients who die,^23^ and ICU use is estimated to increase hospitalization costs by one-third, resulting in a cost difference of $2703 between patients who do and do not use the ICU during a terminal hospitalization.^24^ With these estimates, a decrease in ICU use of 3.5% would translate to a difference in cost of approximately $265 million. Although this provides some understanding of the implications of palliative care implementation for the US health care system, it is important to qualify that these data should not be taken to mean that decreasing ICU use would result in higher value care. Given that ICU use at the end of life is common, and recent studies question whether quality care at the end of life should be equated with less-intense care,^25,26^ it may be that the ICU does provide value to patients and families in a manner that is incompletely understood.

In stratified analyses, we observed that the association of implementing hospital-based palliative care was not consistent across different types of hospitals, with significant associations confined to teaching and large hospitals. Furthermore, in these hospitals, implementing palliative care was also associated with a significant decrease in the number of days in the ICU for patients admitted to the ICU, suggesting that the association extended beyond simply preventing admission to the ICU. In a previous study,^11^ we also observed such heterogeneity of associations, with availability of hospital-based palliative care associated with an increase in discharge to hospice for critically ill patients only in teaching and large hospitals. Reasons for this heterogeneity are unclear, in particular, whether it is associated with differences between palliative care programs, differences between hospital environments and cultures, or both. Palliative care teams are known to have substantial differences in their operational characteristics, such as the amount of staffing and the composition of the palliative care team.^27,28,29^ These differences may be associated with a palliative care program’s effectiveness, because increased staffing has been associated with greater penetration of the palliative care service (defined by the number of consultations seen over the number of total admissions) and a shorter time to initial consultation.^29,30^ Also, hospital cultures may follow different practices, particularly with respect to the use of life-sustaining therapies and delivery of end-of-life care.^31,32,33^ However, in the current study, we lack further detailed data about palliative care programs or hospital practices and, thus, are unable to assess how these factors may have contributed to the observed outcomes.

Our findings are in line with those of prior studies^34,35,36,37,38^ demonstrating that the use of palliative care consultation is associated with a decreased likelihood of patients’ dying in the ICU and shorter ICU stays for patients who died during hospitalization. In contrast to these prior studies, which examined the use of consultation in hospitals with existing palliative care programs, we used a quasi-experimental and ecological approach (the difference-in-differences analysis) to better estimate the expected association between implementing a hospital-based palliative care program and outcomes. Furthermore, we used a large sample of hospitals with varying characteristics that allowed examination of how the outcomes associated with implementing a palliative care program may differ across different types of hospitals and enhanced the generalizability of our results. Prior studies^39,40,41^ of proactive case-finding approaches to increase delivery of palliative care have also found an association between palliative care use and a decrease in the use of specific nonbeneficial life-sustaining therapies, which we did not observe. This may be because the association between palliative care consultation and reductions in the use of such therapies is likely dependent on the timing of initiation^42,43^; in 1 study,^44^ the association between palliative care consultation and ICU costs was not significant unless the consultation was initiated within 2 days of hospital admission.

In combination with other studies, our findings suggest that the availability of palliative care consultation may reduce treatment intensity at the end of life. Less-medicalized death has been associated with higher ratings of quality of life, quality of death, and quality of end-of-life care^45,46,47^ and fewer psychological symptoms in bereaved caregivers, particularly for patients with advanced cancer.^48,49^ Given these data, implementing palliative care programs may be a way to improve the quality of end-of-life care for some patients who die in the hospital.

### Limitations

The main limitation of this study is the possibility of residual confounding; hospitals that chose to implement palliative care programs may be fundamentally different from those that did not. Although we found that hospitals had a decrease in the use of intensive care after implementation of hospital-based palliative care, the overall rate of ICU use was still substantially higher in these hospitals than in hospitals that never implemented palliative care. Thus, it may be that hospitals that did not implement palliative care already had other mechanisms in place to avoid the use of high-intensity interventions or different practices surrounding care at the end of life. We addressed the possibility of hospital-level confounding by including multiple hospital characteristics, accounting for differences in baseline outcomes using a random-effects model, and by conducting a difference-in-differences analysis to account for secular trends. However, it is still possible that other differences in the care provided between hospitals could account for our findings. We were unable to account for differences in actual use of palliative care consultation within hospitals, because this is not accurately captured within administrative data.^12^ We also lacked the granularity of data to be able to confirm the mechanisms underlying our findings, including characteristics of the palliative care team within hospitals, such as the staffing and availability of the team, as well as details about end-of-life care delivered or whether patients had advance directives in place at the time of hospital admission. Although we performed a secondary analysis adjusting for the number of ICU beds at the hospital, true ICU bed availability is often dependent on other factors that were not available to us, such as details of nursing staff and occupancy. We had to exclude most hospitals because they had existing palliative care programs, thus reducing the internal validity of our results. Also, New York is a state with relatively high treatment intensity compared with other states, and these results may not be generalizable to other regions of the United States or other countries.^50^ In addition, we used a decedent cohort, because quality indicators for end-of-life care focus on the intensity of care provided in the immediate period preceding a death. However, prospectively, it is unclear which patients will die, and the impact of the introduction of palliative care on patients with nonterminal conditions was not assessed.

## Conclusions

These data indicate that implementing palliative care programs is associated with a modest decrease in the use of intensive care during terminal hospitalizations and that the association may differ according to hospital characteristics. Future work should focus on identifying characteristics associated with the effectiveness of palliative care programs in decreasing treatment intensity.

## Notes

Supplement. **eAppendix.** Supplemental Methods **eTable.** Difference-in-Differences Analysis Examining the Effect of Implementing Hospital-Based Palliative Care on Resource Utilization During Terminal Hospitalizations, Adjusting for Number of Intensive Care Unit Beds **eFigure.** Parallel Trends Assumption for Unadjusted Outcomes
