# Supplementary material for: Association Between the Implementation of Hospital-Based Palliative Care and Use of Intensive Care During Terminal Hospitalizations
Source: JAMA Netw Open. 2020 Jan 8;3(1):e1918675. doi: 10.1001/jamanetworkopen.2019.18675 (PMC6991248; doi:10.1001/jamanetworkopen.2019.18675)
Supplement: Supplement. — eAppendix. Supplemental Methods eTable. Difference-in-Differences Analysis Examining the Effect of Implementing Hospital-Based Palliative Care on Resource Utilization During Terminal Hospitalizations, Adjusting for Number of Intensive Care Unit Beds eFigure. Parallel Trends Assumption for Unadjusted Outcomes [file jamanetwopen-3-e1918675-s001.pdf]

Supplementary Online Content

Hua M, Lu Y, Ma X, Morrison RS, Li G, Wunsch H. Association between the implementation of hospital-based palliative care and use of intensive care during terminal hospitalizations. *JAMA Netw Open*. 2020;3(1):e1918675. doi:10.1001/jamanetworkopen.2019.18675

eAppendix. Supplemental Methods
eTable. Difference-in-Differences Analysis Examining the Effect of Implementing Hospital-Based Palliative Care on Resource Utilization During Terminal Hospitalizations, Adjusting for Number of Intensive Care Unit Beds
eFigure. Parallel Trends Assumption for Unadjusted Outcomes

This supplementary material has been provided by the authors to give readers additional information about their work.

© 2019 Hua M et al. *JAMA Network Open.*

eAppendix. Supplemental Methods

Detailed Description of Variables Available for Adjustment in Regression Models

Patient-level covariates that were available within SPARCS included age, gender, race (White, Black, other), insurance (private, Medicare, Medicaid, self-pay, other), urban residence, patient type (non-surgical, surgical), number of Elixhauser comorbidities (0, 1-3, ≥4 comorbidities), risk of mortality for the hospitalization (minor, moderate, major, extreme) and presence of sepsis (using the Angus definition). The risk of mortality indicator is calculated using a proprietary grouping software developed by 3M Health Information Systems, and is based on age, comorbidities, procedures and principal diagnosis for the hospitalization. Patients were classified as non-surgical or surgical based on their primary admission DRG for the index admission using DRG groupings for year that the hospitalization occurred (available at www.cms.gov).

Hospital-level variables were obtained from the American Hospital Association (AHA) Annual Survey from 2008-2014 and merged with the SPARCS data; variables were matched to each hospital for each year. Selected variables included whether hospitals were in an urban location, teaching status (defined as being member of the Council of Teaching Hospitals, or having a residency training program or medical school affiliation), and hospital bed size (small <100 beds, medium 100-399 beds, large ≥ 400 beds). We also included the total number of hospital admissions, total number of surgical procedures, and total number of full-time equivalent doctors and nurses employed by the hospital; for these variables, to account for differences in hospital size, we divided variables by the number of hospital beds. For all covariates, no covariate was missing for more than 1% of patients. Patients missing data for particular covariates were handled using listwise deletion in regression analyses.

Detailed Description of Secondary Outcomes

Procedural outcomes were ascertained using ICD-9 procedure codes according to the following algorithms: mechanical ventilation (96.70, 96.71, 96.72), dialysis (39.95, 54.98).

Details of Difference-in-Differences Analysis

To perform a difference-in-differences analysis that allows for variation in treatment timing (i.e. variation in implementation of hospital-based palliative care) amongst hospitals, the model was specified as follows:

$$Y = \beta_1(\text{case}) + \beta_2(\text{year}) + \beta_3(\text{dt}) + \beta_4(\text{covariates}) + \beta_0 + \varepsilon$$

where case is a variable denoting whether hospitals did or did not implement palliative care (case=1 for a hospital that implemented palliative care, and case=0 for a hospital that did not implement palliative care)
year is a categorical variable for year of admission for a given patient
dt is a variable case*prepost, where prepost is variable that serves as an indicator of the pre-post period in case hospitals and set to 0 for non-case hospitals (dt =1 for a case hospital in the post-implementation period and dt=0 otherwise)

In this model, the difference-in-differences estimator is equal to β_3, the coefficient for the dt variable.

Details of Secondary Analyses

For the metastatic cancer subgroup, we identified patients as having that diagnosis using the "metastatic cancer" coding algorithm from the Elixhauser comorbidity index. For the analysis adjusting for number of ICU beds, we examined the AHA data for each hospital for each year. Hospitals that were missing all years of data were

© 2019 Hua M et al. *JAMA Network Open.*

excluded from this analysis. For hospitals that were missing years of data, but otherwise had consistent data, known values were applied to missing years. For hospitals that had data for only one year, the data for the single year were applied to all missing years. For hospitals with missing data, but whose values were not consistent throughout the study period, if the missing value was in the middle of the study period, the value from the previous year was carried forward; if the missing value occurred at the beginning of the study period, the next year of available data was applied to the missing year.

Examples of how missing data were handled for number of ICU beds at a hospital

	**2008**	**2009**	**2010**	**2011**	**2012**	**2013**	**2014**	**Action taken**
A	-	-	-	-	-	-	-	Excluded
B	20	20	20	-	-	20	20	Known values used for missing years
C	20	-	-	-	-	-	-	Known values used for missing years
D	20	20	-	-	30	30	30	Last year carried forward for missing years
E	-	20	20	30	30	30	30	Next available year used for missing years

Dashes indicate missing data. The numbers “20” and “30” are example number of ICU beds.

© 2019 Hua M et al. *JAMA Network Open.*

eTable. Difference-in-Differences Analysis Examining the Effect of Implementing Hospital-Based Palliative Care on Resource Utilization During Terminal Hospitalizations, Adjusting for Number of Intensive Care Unit Beds

Outocmes	Primary Analysis				
	Unadjusted Outcomes			Difference-in-Differences Estimator* (95% CI)	P value
	Implementation of Hospital-Based Palliative Care				
	Yes (N =36,453)		No (N =35,053)		
	Before	After			
Primary Outcome					
ICU use, %[a]	53.3	49.4	45.8	0.90 (0.85–0.95)	<0.001
Secondary Outcomes					
Dialysis, %[c]	9.5	8.8	7.0	0.90 (0.79–1.03)	0.12
Length of Stay, median (IQR)[d]	7 (2–15)	6 (2–13)	6 (2–13)	1.03 (1.00–1.06)	0.11
Mechanical ventilation, %[b,e]	69.1	68.2	59.8	0.97 (0.92–1.01)	0.15
ICU bed utilization days, median (IQR)[d,e]	4 (2–10)	4 (2–9)	4 (1–8)	0.96 (0.92–1.01)	0.12

CI, confidence interval; IQR, interquartile range; ICU, intensive care unit.
[a] This column reports the relative risk, odds ratio or incidence rate ratio as appropriate. All models are adjusted for age, gender, race, type of insurance, urban residence, risk of mortality during hospitalization, sepsis, year of admission, and hospital characteristics including teaching hospital, hospital bed size, total admissions per year/total number of beds, total number of surgical operations performed/total number of beds, full-time equivalent physicians/total number of beds, ICU volume and number of ICU beds.
[b] Results of multilevel robust Poisson regression, with hospital as a random effect.
[c] Results of multilevel logistic regression, with hospital as a random effect.
[d] Results of multilevel negative binomial regression, with hospital as a random effect.
[e] Only includes patients who died in ICU.

© 2019 Hua M et al. *JAMA Network Open.*

eFigure. Parallel Trends Assumption for Unadjusted Outcomes

A.

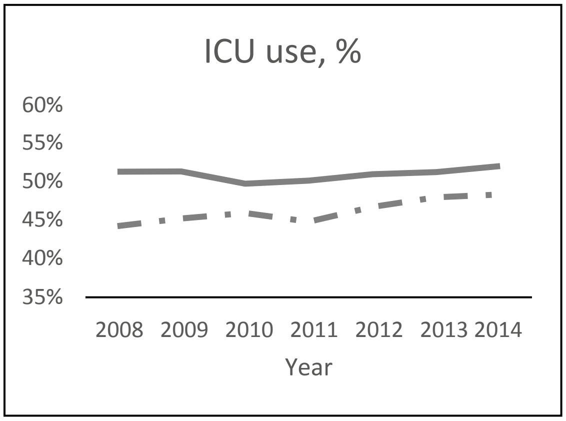

B.

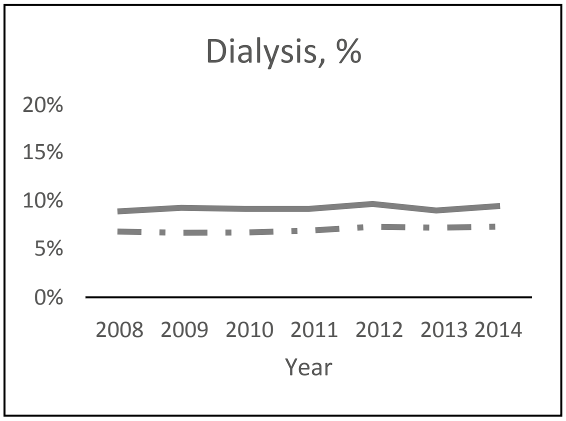

C.

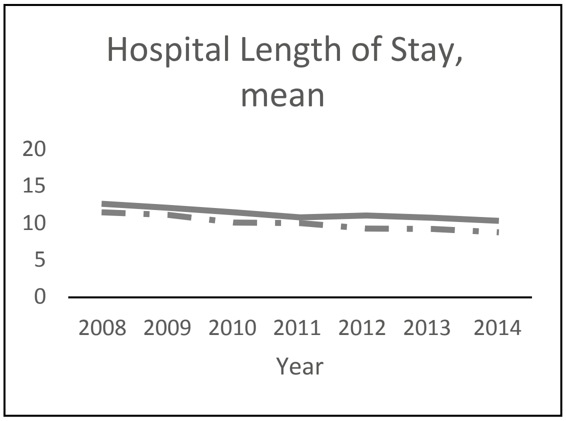

D.

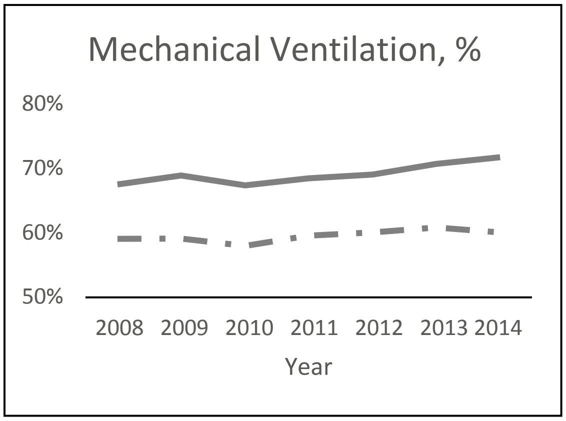

E.

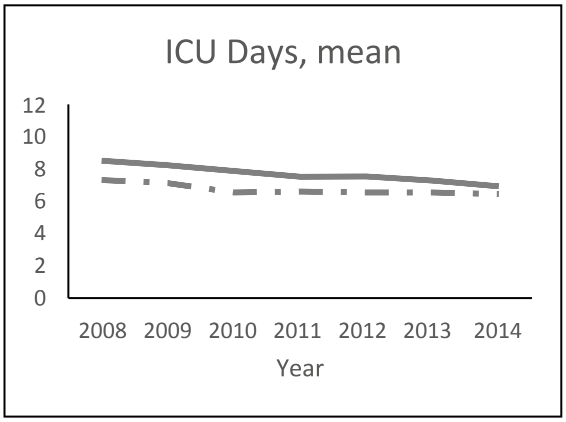

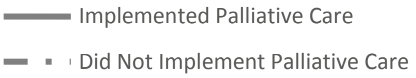

© 2019 Hua M et al. *JAMA Network Open.*
